# Quantification of Exciton Fine Structure Splitting in a Two-Dimensional Perovskite Compound

**DOI:** 10.1021/acs.jpclett.2c00942

**Published:** 2022-05-13

**Authors:** Katarzyna Posmyk, Natalia Zawadzka, Mateusz Dyksik, Alessandro Surrente, Duncan K. Maude, Tomasz Kazimierczuk, Adam Babiński, Maciej R. Molas, Watcharaphol Paritmongkol, Mirosław Mączka, William A. Tisdale, Paulina Płochocka, Michał Baranowski

**Affiliations:** † Department of Experimental Physics, Faculty of Fundamental Problems of Technology, Wroclaw University of Science and Technology, 50-370 Wroclaw, Poland; ‡ Institute of Experimental Physics, Faculty of Physics, University of Warsaw, 02-093 Warsaw, Poland; ¶ Laboratoire National des Champs Magnétiques Intenses, EMFL, CNRS UPR 3228, Université Toulouse, Université Toulouse 3, INSA-T, Toulouse 31400, France; § Department of Chemical Engineering, Massachusetts Institute of Technology, Cambridge, Massachusetts 02139, United States; ∥ Department of Chemistry, Massachusetts Institute of Technology, Cambridge, Massachusetts 02139, United States; ⊥ Institute of Low Temperature and Structure Research, Polish Academy of Sciences, 50-422 Wrocław, Poland

## Abstract

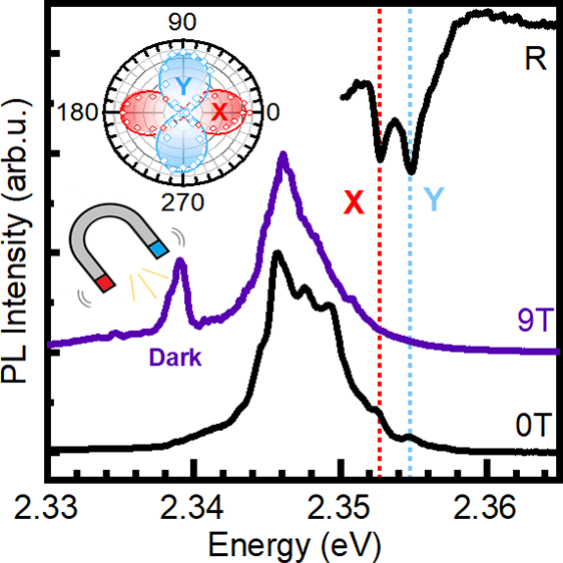

Applications of two-dimensional (2D) perovskites have significantly outpaced the understanding of many fundamental aspects of their photophysics. The optical response of 2D lead halide perovskites is dominated by strongly bound excitonic states. However, a comprehensive experimental verification of the exciton fine structure splitting and associated transition symmetries remains elusive. Here we employ low temperature magneto-optical spectroscopy to reveal the exciton fine structure of (PEA)_2_PbI_4_ (here PEA is phenylethylammonium) single crystals. We observe two orthogonally polarized bright in-plane free exciton (FX) states, both accompanied by a manifold of phonon-dressed states that preserve the polarization of the corresponding FX state. Introducing a magnetic field perpendicular to the 2D plane, we resolve the lowest energy dark exciton state, which although theoretically predicted, has systematically escaped experimental observation (in Faraday configuration) until now. These results corroborate standard multiband, effective-mass theories for the exciton fine structure in 2D perovskites and provide valuable quantification of the fine structure splitting in (PEA)_2_PbI_4_.

An exciton, a quasi-particle composed of an electron and a hole bound by the Coulomb interaction, represents the lowest electronic excitation in a perfect semiconductor. The excitonic states are additionally affected by the exchange interaction (which couples the spins of the electron and hole) and the crystal field, which leads to the so-called fine structure splitting (FSS).^1−4^ These interactions lift the degeneracy of the states with different angular momenta, splitting the bright and dark exciton states. The energy separation and ordering of the exciton states can have a dramatic impact on the optoelectronic properties of materials.^5,6^ The lowest dark exciton states can provide an efficient channel for nonradiative recombination, while whether or not the bright state splits is crucial for advanced quantum devices (single photon sources, entanglement photon sources, or quantum teleportation) and spintronics.^6−15^

Exciton FSS has been the subject of intense investigation for many years,^3,6,16,17^ mostly in 0D systems such as quantum dots and nanocrystals, where quantum confinement enhances the splitting while the broken structural symmetry allows for a control of the degeneracy of the states. Moreover, the observation of the FSS ladder^3,4,18^ provides robust benchmarks to validate band structure models, since it is a product of the band structure, the symmetry of the lattice, and quantum confinement.

The recently exploding field of two-dimensional organic inorganic halide perovskites (2DP) provides a new playground to investigate the exciton FSS and its possible exploitation.^14,18−22^ Due to the quantum and dielectric confinement, the Coulomb interaction enhances the FSS far more than in conventional (epitaxial) low dimensional systems. The splitting of the excitonic states can be as high as tens of millielectronvolts,^19−21^ orders of magnitude larger than in epitaxial structures or nanocrystals.^3,6,16,17^ The large splitting, together with the good optical properties of 2DP, and the simple engineering of band structure and quantum confinement^23−29^ constitute an excellent platform for the investigation of exciton FSS physics.

Surprisingly, despite all of these advantages, there remains controversy regarding the observed optical response, compared to the expected FSS. For example, the reported number of transitions observed in photoluminescence (PL), ascribed to bright in-plane excitonic transitions,^20,30^ exceeds the theoretically predicted number, while the reported values of the bright–dark splitting are far from consistent.^18,19,21^ Clearly, the excitonic fine structure still requires precise quantification. This is especially important for 2D perovskites where the exciton FSS determines the light emission efficiency, and the exciton structure can be controlled by many different independent parameters.^23^

Here we employ low temperature (4.2–10 K) PL and reflectance spectroscopy in a magnetic field to reveal the exciton fine structure of (PEA)_2_PbI_4_ (here PEA is phenylethylammonium) single crystals. We observed a 2.1 ± 0.1 meV splitting of two linearly, and orthogonally, polarized bright in-plane states. We show that the *additional* peaks, often observed in the PL response, are rather related to the exciton–polaron emission which derives from the bright in-plane state. Our results can be elegantly explained in light of the FSS prediction, based solely on symmetry considerations,^18,33,34^ without the need to introduce additional exciton states. Using a magnetic field and a high numerical aperture objective, we measure the PL emission related to the dark exciton state in the Faraday configuration. Our work fulfills the picture of the exciton fine structure in (PEA)_2_PbI_4_. It also provides a starting point for the future engineering of the exciton FSS, for example by tuning the quantum well thickness or the choice of organic spacer, with important implications for spintronic and light emission applications of 2DP.

We have investigated two different (PEA)_2_PbI_4_ single crystals, grown by two very different methods, in order to reduce the possible influence of material imperfections on our findings. The first crystal was grown by the cooling induced crystallization method,^35,36^ and the second one was prepared by slow evaporation of a solvent at room temperature (see Methods for details). Our results are both qualitatively and quantitatively the same for both types of crystals. The results presented here correspond to the crystal grown by the first method (see also Figure S1). The result for the crystal grown by the second method can be found in the Supporting Information (SI; Figures S2 and S3).

Figure 1(a) shows the lattice structure of the investigated (PEA)_2_PbI_4_ crystal. 2DP consists of slabs of octahedral units separated by long organic chains. The planes of the metal-halide octahedra create a quantum well for carriers, while the quantum and dielectric confinement is induced by the organic spacers^24^ as shown in panel (b).

**Figure 1:**
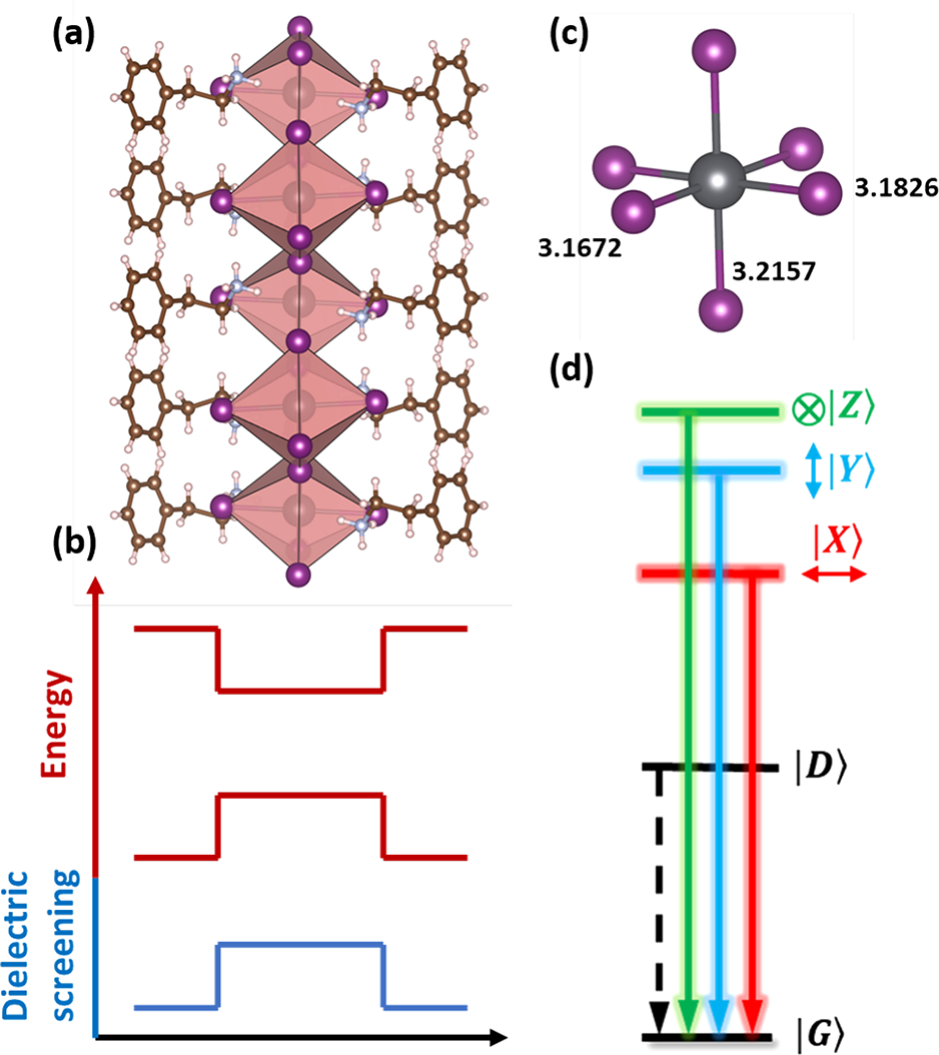
(a) Schematic of the (PEA)_2_PbI_4_ crystal structure.^31^ (b) Schematic of band edge energy profiles and dielectric screening resulting in quantum and dielectric confinement. (c) Detailed view of the octahedral unit with Pb–I bond length.^31,32^ (d) Ladder of excitonic states expected for (PEA)_2_PbI_4_.

The theoretically expected exciton fine structure in metal-halide perovskites (qualitatively the same for 3D and 2D forms) has already been described in the literature.^18,33,34^ Briefly the band structure, together with the lattice symmetry, results in four band edge exciton states characterized by different total angular momenta (*J* = 0 or 1) and their *z*-components (*J*_*z*_ = 0 or ±1). The degeneracy of the exciton states is lifted by the exchange interaction, and the dark singlet (*J* = 0) is the ground state of the system, with three bright states at higher energies. The 2D nature of the crystal naturally distinguishes the *z* axis, lifting the degeneracy of the *J* = 1 states. For the case of equivalent in-plane *x* and *y* axes, there are two degenerate bright states with in-plane dipole moments and *J*_*z*_ = 1 and one bright state with out-of-plane dipole orientation and *J*_*z*_ = 0. The in-plane states couple to circularly polarized light.^18^ However, the crystal structure of (PEA)_2_PbI_4_ belongs to a triclinic system, the in-plane *x* and *y* axes are not equivalent,^37^ and the Pb–I bond lengths in each direction are different (see Figure 1(c)). Therefore, it is expected that the degeneracy of the in-plane states is also lifted.^3,20,33^ The final exciton fine structure is as shown in Figure 1(d), where each of the bright states couples selectively to linearly polarized light along one of the *x*, *y*, or *z* directions. Regarding the presented ladder of exciton states, it is important to emphasize that we adapted it later;^21^ however, there are also reports which situate |**Z**⟩ state below bright in-plane states.^18,22,38,39^

The reflectance spectrum measured in a linear polarization basis, shown in Figure 2(a), is a smoking gun signature of the bright in-plane exciton FSS. For the two selected orthogonal polarizations π_*x*_ and π_*y*_, the free exciton resonances (FX_*x*_ or FX_*y*_) are clearly resolved. The two linearly polarized transitions behave exactly as expected from theory. The measurements were performed using a low numerical aperture objective (NA = 0.55), selectively sensitive to in-plane states. The observed splitting of the bright in-plane exciton states, ≃2.1 ± 0.1 meV, is significantly larger than the reported splitting in 3D perovskites.^40^ This is expected due to the significantly higher exciton binding energy and the enhanced overlap of electron and hole wave functions in 2D perovskites, which enhance the exchange interaction.^3,4,34^ To further corroborate that the reflectance response is composed of two linearly polarized transitions in Figure 2(b), we show the full dependence of the reflectance spectrum versus the detection polarization angle. The intensity and shape of the spectrum exhibit an oscillatory behavior which is characteristic for two split and orthogonally polarized transitions, when we detect a varying contribution from each state as the detection polarization analyzer is rotated.

**Figure 2:**
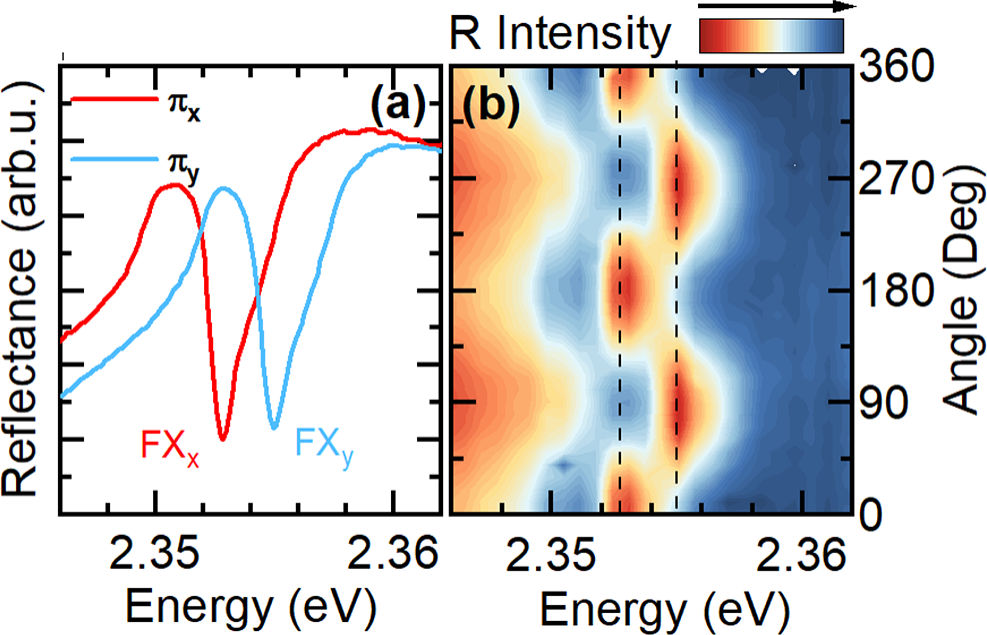
(a) Reflectance spectrum measured in two orthogonal linear polarizations showing clear splitting. (b) Dependence of the reflectance spectrum versus polarization angle.

The PL response exhibits a more complex spectrum, as is shown in Figure 3. It is dominated by a multiple peak structure which is red-shifted with respect to the excitonic resonances visible in the reflectance response (violet line). Here the spectra were collected without polarization optics, so both in-plane transitions are observed simultaneously. On the high energy shoulder of the PL band we can distinguish two weaker PL features (shaded area in Figure 3). The separation and position of these peaks correspond exactly to the excitonic transitions FX_*x*_ and FX_*y*_ visible in the reflectance spectrum; that is, they result from the in-plane free excitons’ recombination. To confirm the origin of these transitions, we determine their selection rules by means of polarization resolved measurements.

**Figure 3:**
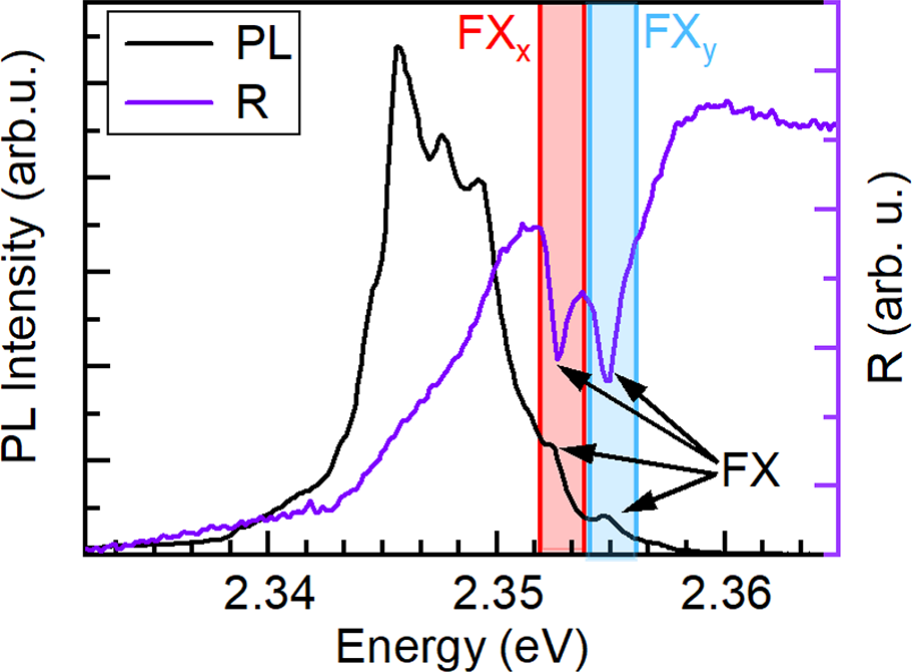
PL (black) and reflectance (violet) response of (PEA)_2_PbI_4_ single crystal in the backscattering geometry; the shaded areas indicate FX_*X*_ and FX_*Y*_ transitions.

Figure 4(a and b) shows that the two high energy PL features are linearly polarized. For two orthogonal polarizations π_*x*_ and π_*y*_ the higher or lower energy FX transitions are suppressed (see Figure 4(a)). Naturally, the lower energy (FX_*x*_) transition is more intense due to the thermal distribution of excitons over the two in-plane states. Our analysis (see Figure S4 in the SI) of the FX PL intensity displays a characteristic double lobe shape for each state, in the polar coordinate plot shown in Figure 4(b). The intensity of both peaks is well fitted with sin^2^(ϕ + δ) (ϕ is the detection analyzer angle, and δ is a phase), corroborating its linear polarization, and the phase difference for two peaks is 90°, showing their orthogonality.

**Figure 4:**
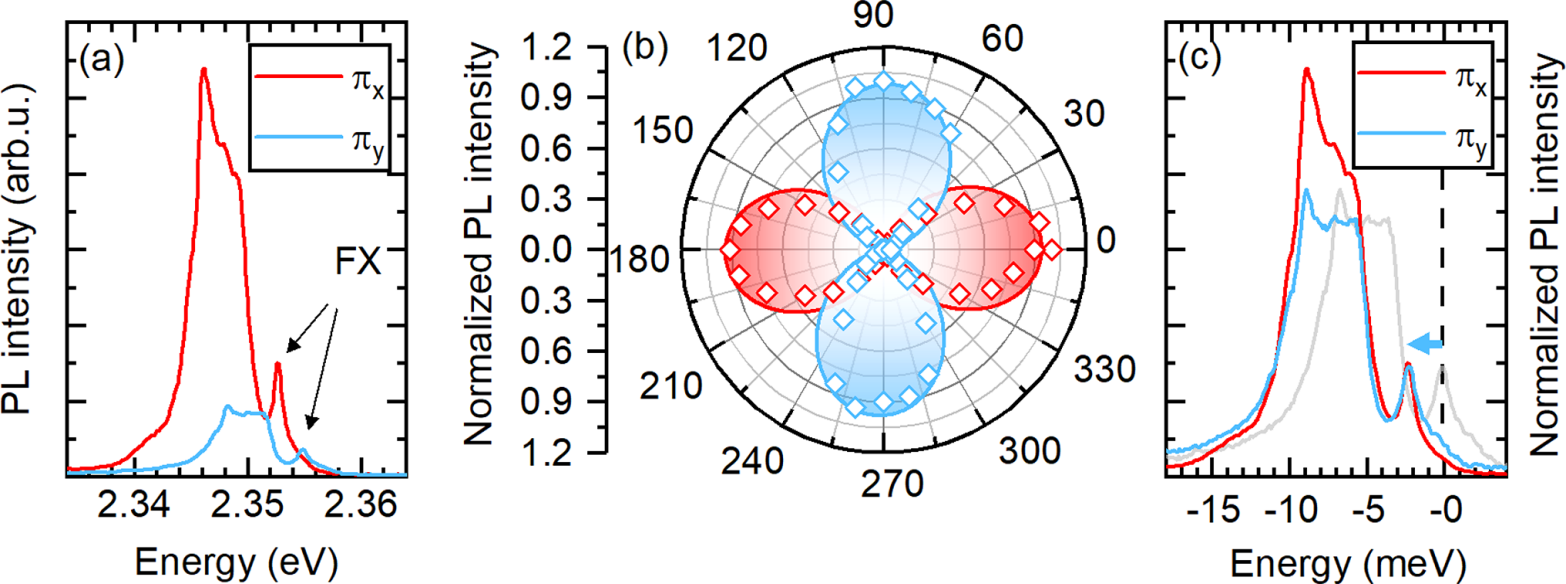
(a) PL spectra measured for two orthogonal polarizations. (b) Polar plot of the FX_*X*_ and FX_*Y*_ transitions; PL intensity as a function of polarization detection angle. (c) PL spectra measured in two orthogonal polarizations. The spectra are plotted relative to FX_*Y*_ transitions (indicated by dashed lines). The light gray line is a PL spectrum measured in π_*Y*_ polarization. The blue line represents the spectrum measured in π_*Y*_ polarization shifted to the low energy side by the value of the bright in-plane fine structure splitting (2.1 meV). It shows good overlap with the spectrum measured in the second polarization (red line).

It is important to note that the dominant red-shifted PL bands have the same linear polarization as the FX transitions. It clearly blue-shifts and loses intensity when the detection polarization changes from π_*x*_ to π_*y*_. Unfortunately, the partial overlap of the emission bands precludes a detailed investigation of intensity versus detection polarization angle. It is significant that there are no corresponding transitions in the reflectance response (see Figure 3) either at the same energy or on the high energy side of the FX transitions (as expected for phonon replicas).^41^ We hypothesize that the dominant PL emission is linked to a reconfiguration of the soft and the ionic perovskite lattice.^42−44^ The lattice deformation (phonon cloud) dresses the free exciton states, leading to a complex exciton–polaron landscape representing specific lattice reorganizations.^45−48^

Our results show that the different exciton–polaron states (forming a multiple-peak, red-shifted PL response) maintain the selection rules and fine structure of the bare bright in-plane free exciton states. Indeed, the intensity and energy position of the dominating PL band evolve qualitatively in the same way as for the FX states. For π_*y*_ polarization all PL features are blue-shifted by ≃2.1 meV with respect to π_*x*_ polarization and are clearly less intense. At the same time the shape (structure) of the PL spectrum is maintained for both polarizations. These observations are summarized in Figure 4(c), where the spectrum measured in π_*y*_ polarization (normalized to equalize the FX PL intensities) is shifted to lower energy by ≃2.1 meV. Clearly, the spectra measured in two orthogonal polarizations possess the same features and broadening, showing that the states responsible for the inherent properties of the dominant PL response are the free in-plane excitonic transitions. Crucially, this hypothesis avoids the need to introduce additional excitonic states^20^ to explain the PL spectrum, and it is in agreement with most basic exciton models derived from symmetry considerations.^18,33,39^ It is worth noting that in the light of our results, the common assignment of the PL maximum to the free exciton transition^49−52^ should be reconsidered, since the dominating PL response results from the inherent polaronic character of the excitons in metal-halide perovskites^42,47^ and free exciton transitions. In other words, the dominating PL band at low temperature results from excitonic states already “dressed with phonons”. The excellent crystalline quality of the investigated samples results in a reduced broadening of the transitions which allows us to disentangle these two contributions. A precise knowledge of the free exciton transition energy is crucial to correctly determine the FSS from PL spectra, e.g. bright–dark splitting.^14,19,21^

To access the dark state, we use magneto-optical spectroscopy^21,53,54^ in the Faraday configuration (Figure 5(a)) with **B**∥**k**∥*c* (where **B** is the magnetic field vector and **k** is the light wave vector). The magnetic field mixes the dark exciton state with the out-of-plane state.^54^ Mixed exciton states, in particular the brightened dark state in the Faraday configuration, can be expressed as a linear combination of zero magnetic field states and in the chosen basis take following form,
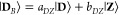
1where the *a*_*DZ*_ and *b*_*DZ*_ coefficients depend on **B** and the energy separation of the dark and out-of-plane exciton states.^53,54^ The transfer of oscillator strength to the dark state from the |**Z**⟩ exciton state, together with the use of the high numerical aperture objective (here NA = 0.82) and the preferential occupation of the dark state at low temperatures, allows us to reveal the signature of the dark states in the PL response. Figure 5(b) shows PL spectra measured with the sample placed in different magnetic fields. With increasing magnetic field, a new sharp feature appears on the low energy side of the dominant PL band, which we attribute to the brightened dark exciton state. The intensity of this new transition increases quadratically with magnetic field (see Figure 5(c), which is characteristic for a dark state brightened due to mixing with the bright exciton state in the weak field limit.^55,56^ The dark state is separated by ∼15.5 ± 1.0 meV from the average bright in-plane excitonic state’s energy. This value is lower but still in reasonable agreement with the splitting determined recently for the 2DP thin films in the Voigt configuration (21.6 ± 3.3 meV).^26^

**Figure 5:**
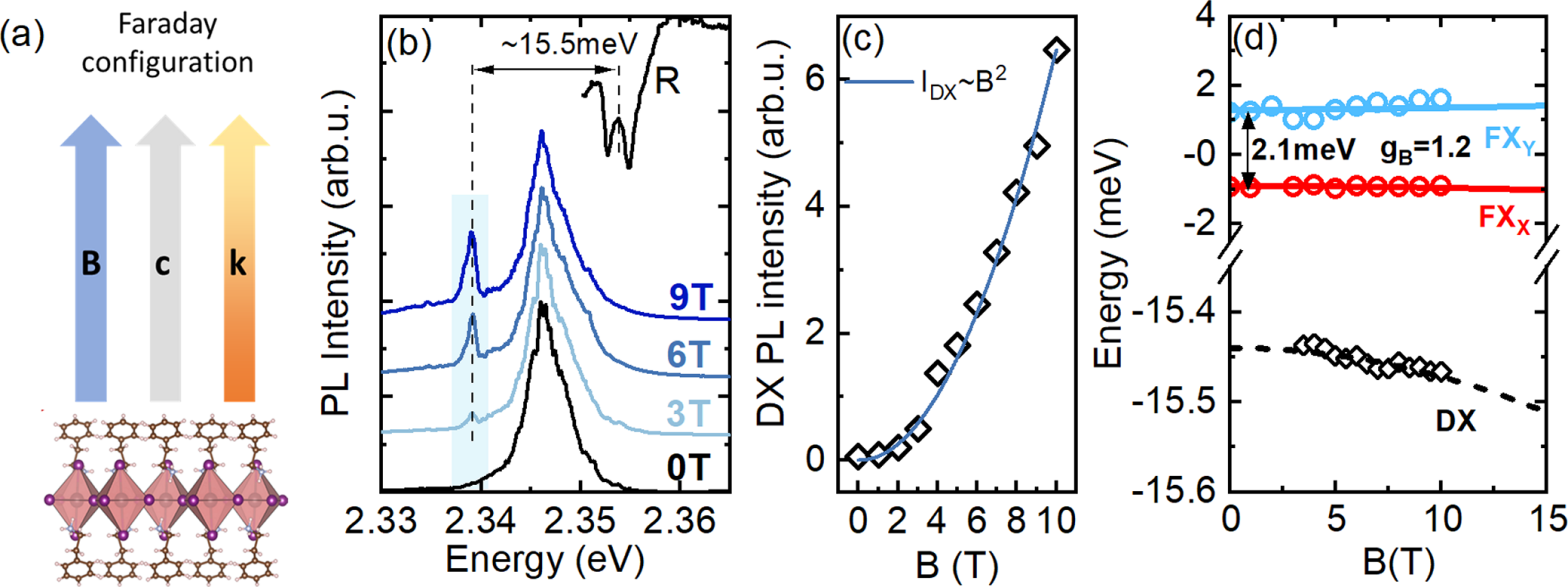
(a) Scheme of the Faraday configuration used in measurements with the magnetic field vector **B** parallel to the light field vector and **k** crystal out-of-plane direction *c*. (b) PL spectra measured under the magnetic field. The light blue shading indicates the position of the brightened dark state. The arrow indicates the distance between the in-plane bright states and the dark exciton state. (c) Dark exciton transition PL intensity versus magnetic field showing quadratic dependence. (d) Summary of exciton states’ shifts in magnetic field (symbols) together with lines according to eq 2 for bright states. The dashed line for dark exciton state (DX) is a guide for the eye. The energy shifts of tow bright exciton states FX_Y_ and FX_X_ are extracted from the reflectance spectrum, presented in Figure S5 in the SI, while the DX energy is based on the PL spectra.

In the Faraday configuration the magnetic field induced shifts of two pairs of coupled states (|**X**⟩, |**Y**⟩ and |**Z**⟩, |**D**⟩) were described by the following formula:^53,54^
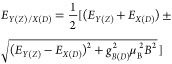
2where *B* is the magnetic field, μ_B_ is the Bohr magneton, and *g*_*B*(*D*)_ is the bright (dark) exciton *g*-factor. The energy shifts in magnetic field of two bright in-plane states and the dark state are summarized in panel (d) of Figure 5. The two in-plane bright states’ shifts are extracted from the magnetoreflectance spectrum, where they are better resolved (see Figure S5), while the position of the dark state is obtained from the fitting of the corresponding PL peak with the Lorentz function (see Figure S6). As is shown by the solid blue and red line, the shifts of bright in-plane state can be well described with the X-Y splitting of 2.1 meV and *g*_*B*_ = 1.2 ± 0.1 taken after high field investigations.^21,26^ The PL peak ascribed to the dark state exhibits a slight red-shift with increasing magnetic field, as expected for the lowest lying dark state,^53,54^ corroborating our assignment. Unfortunately, even with the use of a high NA objective, we do not observe a clear feature related to the Z state, in either PL or reflectance spectra. Therefore, we cannot draw a conclusion about its energy position with respect to other states, which also prevents us from determining the dark state *g*-factor in the Faraday configuration.

In conclusion, we have reported a splitting of bright in-plane exciton states (2.1 ± 0.1 meV) in the reflectance and PL response of (PEA)_2_PbI_4_ single crystals. We show that the multiple peak structure observed in the PL response is probably related to exciton–polaron emission, which inherits selection rules from the free bright exciton states. With the use of a magnetic field, we have, for the first time, brightened the dark exciton state in 2D perovskites in the Faraday configuration, quantifying the bright–dark and in-plane state splitting corroborating the exciton picture resulting from multiband, effective-mass models (up to the uncertainty of the Z state position). In this way we have provided a quantitative picture of the exciton structure of the (PEA)_2_PbI_4_ 2D perovskite, providing a firm base for future investigation of the exciton FSS and spintronic applications of 2DP.

## Methods

*Synthesis and Sample Preparation*. The first method was cooling-induced crystallization performed by following the previously reported procedure.^35,36^ A mixture of lead(II) oxide (PbO, 0.558 g, 2.5 mmol), phenethylamine (198 μL, 2 mmol), and hypophosphorus acid (H_3_PO_2_, 425 μL) was dissolved in 8 mL of 55% hydrogen iodide solution (HI) to form a bright yellow solution at 130 °C. After that, the solution was allowed to cool slowly to room temperature to yield (PEA)_2_PbI_4_ crystals.

In the second method to obtain single crystals of PEA_2_PbI_4_, 2 mmol of PbI_2_ (99.999%, Sigma-Aldrich) was dissolved in 4 mL of concentrated HI (57 wt % in H_2_O, stabilized with H_3_PO_2_, Sigma-Aldrich) and 1 mL of H_3_PO_2_ acid (50 wt % in H_2_O, Sigma-Aldrich) under stirring and heating to 50 °C. In a separate vial, 5 mmol of phenethylamine (0.55 mL, 99.5% Sigma-Aldrich) was added to 6 mL of HI under stirring and then acetonitrile was added until complete dissolution of the sediment. The phenethylamine solution was added to the PbI_2_ solution under stirring, and the hot plate was turned off. Since orange crystals started to grow at room temperature, more acetonitrile was added until a clear solution was obtained. Then, the vial was covered with a paraffin film in which small holes had been created. This vial was left undisturbed at room temperature, and the orange crystals with dimensions up to 5 mm that grew at the bottom of the vial were isolated from the liquid after 1 week.

*Optical Measurements*. For PL and reflectance measurements without the use of a magnetic field, the samples were mounted in the cold finger He flow optical cryostat. All these measurements were performed at 4.2 K. The PL was excited with a 405 nm CW laser. For reflectance measurements the white light was provided by a broad-band halogen high intensity fiber light source (Thorlabs). The excitation and signal collection were done with the use of long working distance, 50× magnification microscope objective with an aperture of 0.55. The optical response was analyzed with a 50 cm long monochromator with a grating of 1200 grooves per mm and detected with a liquid-nitrogen-cooled CCD camera. Magneto-PL was performed at *T* = 10 K in a superconducting magnetic coil in fields up to 12 T. A 515 nm laser and a microscope objective with NA = 0.82 were used. The signal from the magnet was collected by an optical fiber and analyzed with a 75 cm long monochromator with a grating of 1800 grooves per mm and detected with a liquid-nitrogen-cooled CCD camera.

## Supporting Information Available

Optical response of other (PEA)_2_PbI_4_ crystals; reflectance spectra of (PEA)_2_PbI_4_ under a magnetic field; fitting examples (PDF)
