# Supplementary material for: Quantification of Exciton Fine Structure Splitting in a Two-Dimensional Perovskite Compound
Source: J Phys Chem Lett. 2022 May 13;13(20):4463–9. doi: 10.1021/acs.jpclett.2c00942 (PMC9150119; doi:10.1021/acs.jpclett.2c00942)
Supplement: Supplementary file 1 — jz2c00942_si_001.pdf [file jz2c00942_si_001.pdf]

Quantification of Exciton Fine Structure Splitting in a Two-Dimensional Perovskite Compound - Supplementary Information

Katarzyna Posmyk,[†] Natalia Zawadzka,[‡] Mateusz Dyksik,[¶,†] Alessandro Surrente,[†] Duncan K. Maude,[¶] Tomasz Kazimierczuk,[‡] Adam Babiński,[‡] Maciej R. Molas,[‡] Watcharaphol Paritmongkol,[§,∥] Mirosław Mączka,[⊥] William A. Tisdale,[§] Paulina Płochocka,[*,¶,†] and Michał Baranowski[*,†]

†*Department of Experimental Physics, Faculty of Fundamental Problems of Technology, Wroclaw University of Science and Technology, 50-370 Wroclaw, Poland*

‡*Institute of Experimental Physics, Faculty of Physics, University of Warsaw, 02-093 Warsaw, Poland*

¶*Laboratoire National des Champs Magnétiques Intenses, EMFL, CNRS UPR 3228, Université Grenoble Alpes, Université Toulouse, Université Toulouse 3, INSA-T, Grenoble 38042 and Toulouse 31400, France*

§*Department of Chemical Engineering, Massachusetts Institute of Technology, Cambridge, Massachusetts 02139, United States*

∥*Department of Chemistry, Massachusetts Institute of Technology, Cambridge, Massachusetts 02139, United States*

⊥*Institute of Low Temperature and Structure Research, Polish Academy of Sciences, 50-422 Wrocław, Poland*

E-mail: paulina.plochocka@lncmi.cnrs.fr; michal.baranowski@pwr.edu.pl

Results for other crystals and fitting examples

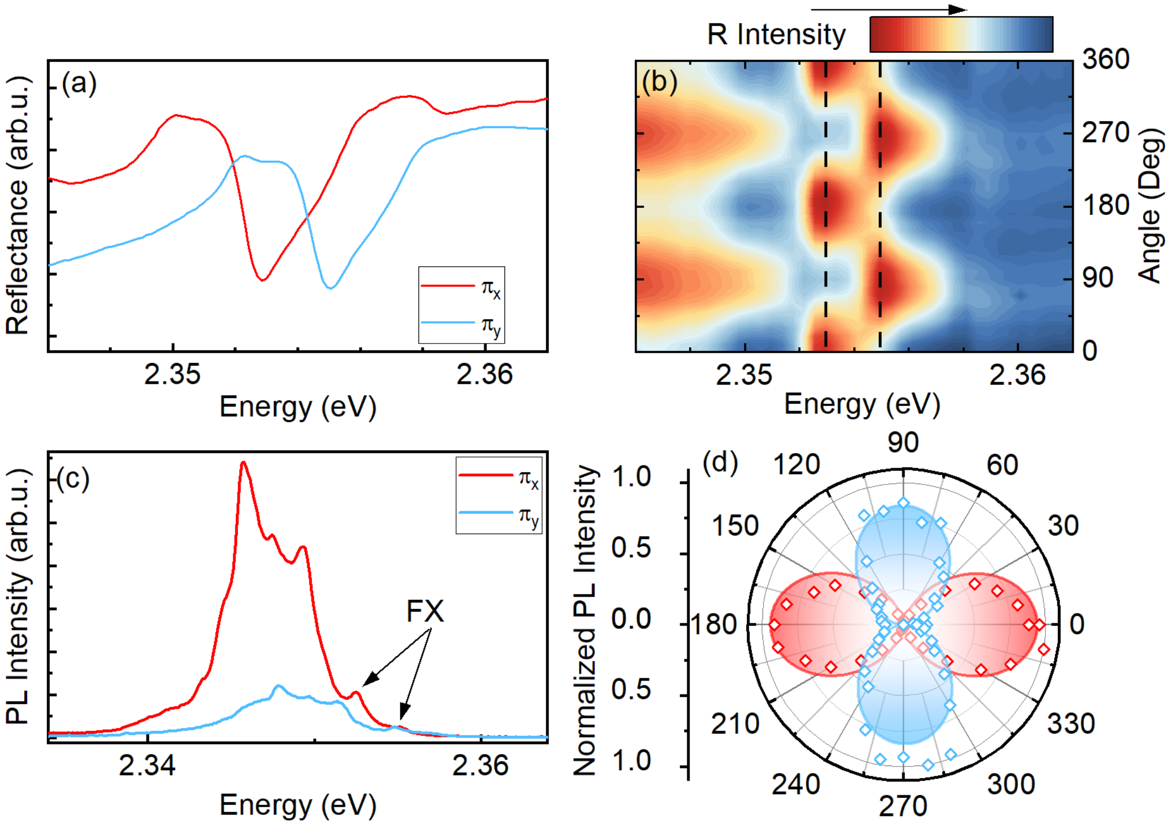

Figure S1: Polarization resolved optical studies of another $(PEA)_2PbI_4$ single crystal grown by cooling induced crystallization method. (a) reflectance spectrum measured in two orthogonal linear polarization. (b) dependence of reflectance spectrum vs polarization angle. (c) PL spectra measured for two orthogonal polarizations. (d) Polar plot of FX_X and FX_Y transitions, PL intensity as a function of polarization detection angle.

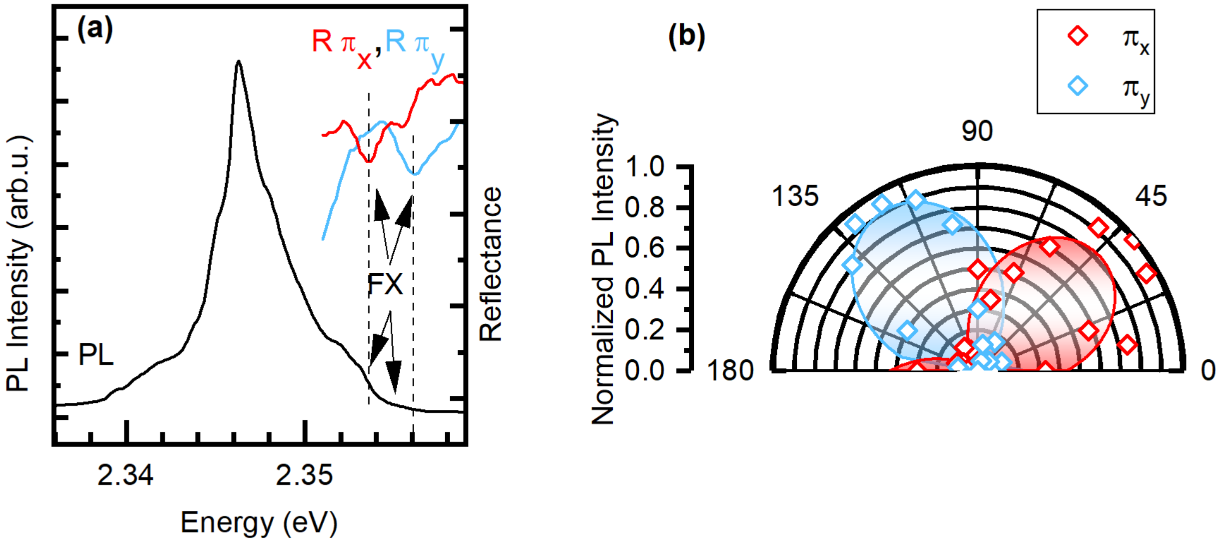

Figure S2: Optical response of $(PEA)_2PbI_4$ single crystal grown by slow evaporation of a solvent method. (a) PL spectra measured without polarization optics (black) and reflectance (red and blue) measured for two orthogonal polarization. (b) Polar plot of FX_X and FX_Y transitions, PL intensity as a function of polarization detection angle.

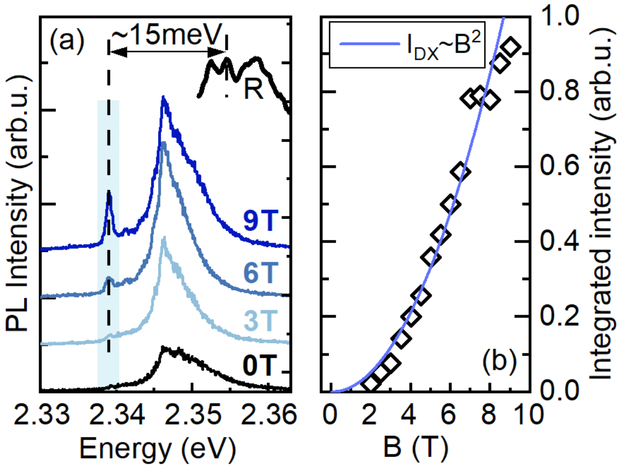

Figure S3: PL response under the megnetic field of $(PEA)_2PbI_4$ crystal grown by slow evaporation of a solvent. (a) PL spectra measured under the magnetic field. The light blue shading indicate the position of brightened dark state. Arrow indicate the distance between in-plane bright states and dark exciton states (b) dark exciton transition PL intensity vs magnetic field showing quadratic dependence.

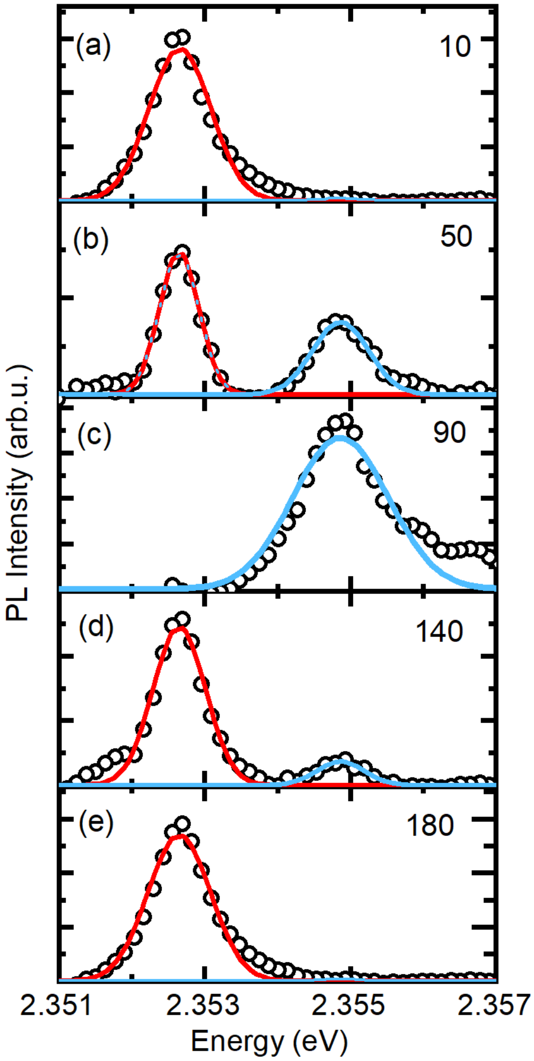

Figure S4: Examples of free excitons PL fitting at different detection polarization angle

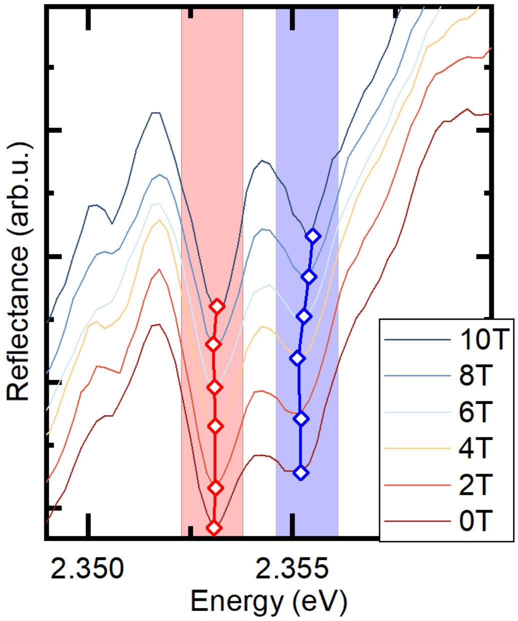

Figure S5: Reflectance spectra for different magnetic fields together with indicated shifts of two transitions.

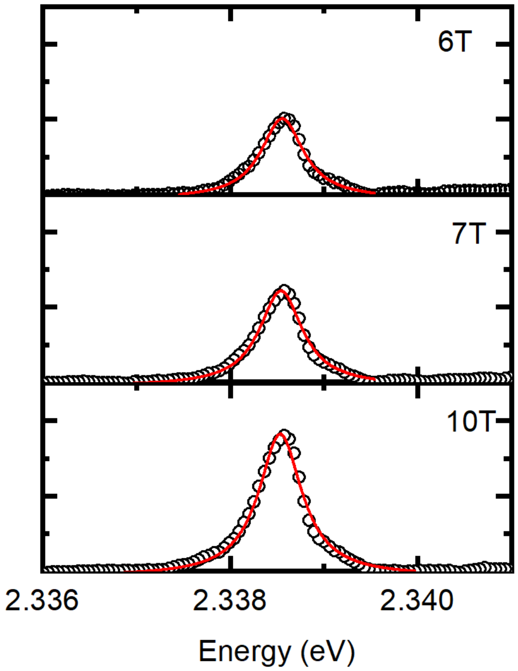

Figure S6: Examples of dark exction PL peak fitting fof different fields. Because the dark state rises on the low energy tail of dominating PL peak before the fitting the straight line was subtracted to exclude it impact.
